# A Case of Primary Multifocal Cutaneous Mucormycosis in a Pediatric Patient with Newly Diagnosed Acute Lymphoblastic Leukemia

**DOI:** 10.3390/medicina59050905

**Published:** 2023-05-09

**Authors:** Denis Niyazi, Milena Belcheva, Stoyan Vergiev, Valeria Kaleva, Temenuga Stoeva

**Affiliations:** 1 Microbiology Laboratory, University Hospital “St. Marina”—Varna, 9010 Varna, Bulgaria; temenuga.stoeva@abv.bg; 2 Medical University of Varna, Varna 9002, Bulgaria; milenabeltcheva@yahoo.com (M.B.); valeria@kalevi.eu (V.K.); 3 Pediatric Hematology and Oncology Clinic, University Hospital “St. Marina”—Varna, 9010 Varna, Bulgaria; 4 Department of Ecology and Environmental Protection, Technical University of Varna, 9010 Varna, Bulgaria; stvergiev@gmail.com

**Keywords:** cutaneous mucormycosis, leukemia, pediatric mucormycosis, primary mucormycosis

## Abstract

Mucormycosis, caused by the widespread molds of the *Mucorales* order, is an insidious infection that manifests in different clinical forms. Even the most benign form, the cutaneous mucormycosis, can present with severe complications and a fatal outcome in patients with a suppressed immune system and underlining comorbidities. We present a rare case of a proven primary multifocal cutaneous mucormycosis in a child with newly diagnosed acute leukemia without multiorgan dissemination. Various laboratory techniques (histopathological, cultural and molecular-genetic) were used to detect and confirm the diagnosis. Etiological therapy (liposomal amphotericin B, 5 mg/kg) combined with surgical intervention were used to manage the infection. The case shows that a rapid and complex diagnostic approach is of crucial importance for the timely initiation of adequate therapy, as well as for the successful management of this life-threatening fungal infection.

## 1. Introduction

Mucormycosis (also known as zygomycosis) is the third most common invasive fungal infection after *Candida* and *Aspergillus* associated infections [1]. Its causative agents are molds belonging to the *Mucoraceae* family of order *Mucorales*, which inhabit the soil and organic matter [2]. Except in the environment, molds can be found in healthy individuals colonizing the upper respiratory tract, as well as the digestive system [2]. The first case of infection caused by these fungi was described in 1885 by Arnold Paltauf, giving it the name Mycosis Mucorina [3]. Years later, in 1957, RD Baker introduced the currently used term “mucormycosis” [4]. While mucormycosis is most frequently caused by *Rhizopus arrhizus* (*Rhizopus oryzae*), it can also occur due to some representatives of the genera *Mucor*, *Lichthemia* (*Absidia*) and *Cunninghamella* [2]. The infection affects different anatomic areas and presents itself in a rhino-orbital-cerebral, pulmonary, cutaneous or an intestinal form [5]. Regardless of its clinical presentation, mucormycosis is a life-threatening infection with a mortality rate of 35–66% [6].

We present a rare case of multifocal cutaneous mucormycosis without dissemination in multiple organs or the central nervous system in a 2-year-old child with newly diagnosed acute lymphoblastic leukemia.

## 2. Case Report

A 2-year-old boy was admitted to the Pediatric Hematology and Oncology ward with fever, drowsiness, decreased appetite and gingivorragia. The symptoms first appeared a week before hospitalization. Ambulatory blood tests revealed severe anemia, thrombocytopenia and lymphocytosis. The patient was in an impaired general condition. A single indurated lesion was found in the left cubital fossa. The child’s family, perinatal, growth, and developmental histories were unremarkable. He had a complete vaccination record for his age.

A bone marrow examination revealed total bone marrow blast cell infiltration. The flowcytometry confirmed the diagnosis of acute B-cell lymphoblastic leukemia. The patient started induction chemotherapy (Methyprednisolone, Vincristine, Epirubicin hydrochloride and PEGylated L-aspargase) according to the ALL-IC BFM 2009 IA protocol along with standard supportive therapy. Laboratory blood test results at admission and during the hospital stay are presented in Table 1.

**Figure 1:**
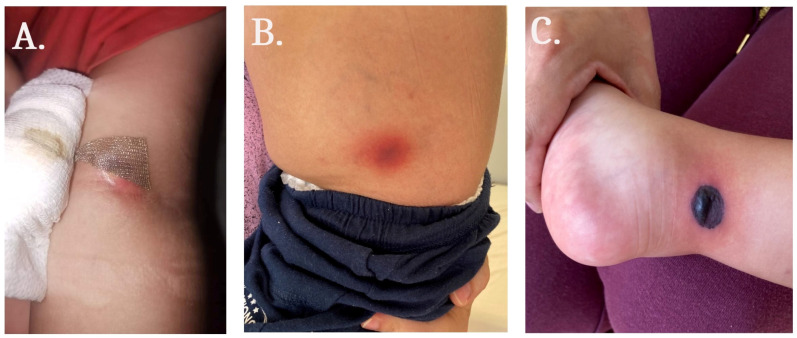
Skin lesions with different anatomic locations: (**A**) left cubital fossa; (**B**) left flank; and (**C**) right leg.

**Figure 2:**
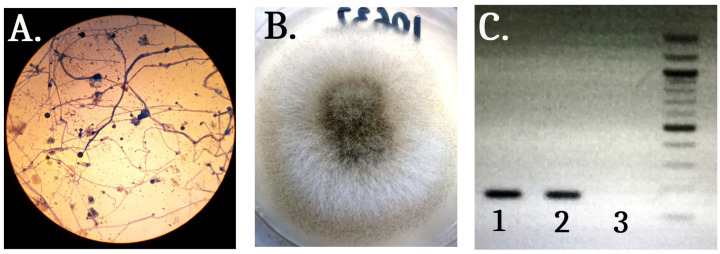
Microbiological examination of the aspirate sample, collected from the skin lesion. (**A**) Microscopic examination by Lactophenol cotton blue, demonstrating nonseptate fungal hyphae forming oval sporangia. (**B**) Grey fluffy mycelium on Sabouraud dextrose agar after 48 h of incubation. (**C**) PCR result: 1—positive biopsy skin sample, PCR product of 124 bp; 2—positive control; and 3—negative control.

During the induction chemotherapy, an increase in the diameter of the infiltrate located in the left cubital fossa and change in its color from red to black was observed (Figure 1). Aspirates from the skin lesions were collected and sent for microbiological examination. After inoculation onto Sabouraud dextrose agar (SDA), a mold forming grey fluffy mycelium, which is suspicious for *Mucor* sp., was observed. Microscopic examination of the samples with lactophenol cotton blue revealed that nonseptate fungal hyphae were forming oval sporangia (Figure 2). Rhizoids and stolons were not observed. A nested Polymerase chain reaction (PCR) was used for 18S rRNA gene detection of clinically relevant agents of mucormycosis in the collected skin samples (*Lichtheimia* spp., *Rhizomucor pusillus*, *Mucor* spp., *Actinomucor elegans*, *Cokeromyces recurvatus*, *Saksenaea* spp., *Apophysomyces* spp., *Rhizopus* spp., *Syncephalastrum racemosum*, *Cunninghamella* spp.) [7]. The following oligonucleotide sequences were chosen for the outer PCR: Mucor1 (5′-WTT ACC RTG AGC AAA TCA GA-3′) and Mucor2 (5′-CAA TCY AAG AAT TTC ACC TCT AG-3′), and for the inner PCR: Mucor3 (5′-AGC ATG GAA TAA TRA AAY A-3′) and Mucor4 (5′-AGC ATG GGA TAA CGG AAT A-3′). The PCR conditions included initial denaturation at 94 °C for 10 min, 35 cycles of denaturation at 94 °C for 30 s, annealing at 55 °C for 30 s, and elongation at 72 °C for 30 s, which was then followed by a final elongation at 72 °C for 10 min. A PCR product of 124 bp was detected after resolution of the PCR amplicons by horizontal gel-electrophoresis, proving mucormycosis.

**Figure 3:**
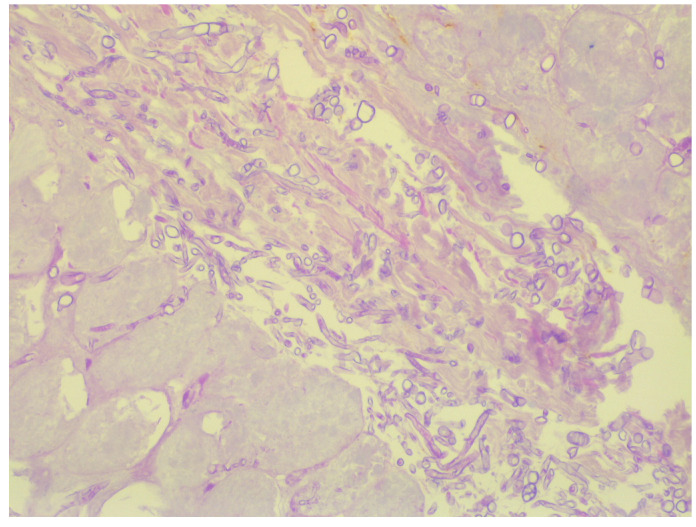
Histological examination demonstrating fatty necrosis, coarse, nonseptate, some branching at right angles of fungal hyphae (hematoxylin and eosin, ×20).

After microbiological identification of the causative agent, liposomal amphotericin B therapy (5 mg/kg) was initiated and surgical intervention was performed on all seven skin lesions. Biopsy samples were sent for microbiological and histological examination. The cultural method confirmed the result from the initial microbiological testing performed before antifungal and surgical treatment. After 48 h of incubation at 30 °C, the fungal growth characteristic of *Mucoraceae* was observed on SDA. The histological examination revealed fatty necrosis, leukocyte infiltrates, an abundance of Periodic acid–Schiff stain (+), coarse, nonseptate, branching at right angles of fungal hyphae, and angioinvasion (Figure 3).

Along with the classical microbiological and histological examinations, as well as PCR, several additional tests were carried out, including the serum galactomannan antigen test, multiple blood cultures, cerebrospinal fluid, urine, throat and nasal cultures, fecal screening for colonization by multidrug-resistant microorganisms, and latent viral infections tests (HBV, HCV, HIV, CMV, EBV). All results were negative. Computed tomography scans of the chest, abdomen, and pelvis did not reveal abnormalities. Apart from the skin lesions, no other primary or secondary source of fungal infection were found during the patient’s hospital stay.

Based on the data obtained from the laboratory tests and using the revised and updated guidelines of the European Organization for Research and Treatment of Cancer and the Mycoses Study Group Education and Research Consortium, a diagnosis of proven primary multifocal cutaneous mucormycosis caused by *Mucor* sp. with no involvement of internal organs or central nervous system was established [8].

After surgical excision and antifungal therapy, healthy granulation of the surgical wounds were observed with no new lesions. Antifungal therapy with liposomal amphotericin B continued for 4 months. Following a re-evaluation, the patient was assessed as successfully cured.

## 3. Discussion

Mucormycosis is an exogenous, life-threatening and opportunistic infection associated with high morbidity and mortality in children and adults [9]. It is characterized by acute and aggressive progression, angioinvasion, subsequent thrombosis and tissue necrosis [10]. The incidence of mucormycosis varies greatly in different parts of the world. It is highest in India with an incidence of 14 per 100,000 individuals, while in Europe and the United States it is much lower with approximately 0.2 and 1.7 cases annually per 100,000 individuals, respectively [11,12]. The reason for the high incidence of mucormycosis in India might be associated with the specific environmental conditions, hot weather and humid climate [13].

Predisposing factors for the development of mucormycosis are hematological malignancies, transplantation, diabetes, iron overload and deferoxamine use [2]. The rhino-orbital-cerebral, pulmonary and intestinal forms are usually associated with the aforementioned risk factors. Cutaneous mucormycosis, which ranges between 10 and 19% of all cases, is caused by fungal spores that enter the dermis after direct skin contact with contaminated materials [14]. This form is rarely associated with an underlying disease and can occur, both in individuals with immunosuppression, and in immunocompetent hosts. Almost half of the cases of cutaneous mucormycosis are not associated with a weakened immune system, but develop after severe trauma (burns and injuries) that severely affect the skin and integrity of the mucous membrane [15]. The frequency of this form increases after natural disasters [2]. Neither burns and trauma (including insect bites) nor natural disasters were considered in the clinical case presented above.

The cutaneous form of mucormycosis manifests with a red and swollen plaque, which can later ulcerate and necrotize. Blood vessels, subcutaneous layers and muscles may also be affected [16]. Upon hospitalization, our patient developed new lesions and infiltration in the cubital fossa rapidly increased after starting chemotherapy. We assume that the chemotherapy-induced neutropenia facilitated the lymphatic dissemination of the molds from the primary lesion to other skin areas.

Authors from different parts of the world (India, Spain and the United States) have described cases of cutaneous mucormycosis in pediatric patients with various risk factors. In concordance with our case, most of them were with hematological malignancies (Hodgkin lymphoma, acute myeloblastic leukemia, aplastic anemia) or had underwent hematopoietic stem cell transplantation, but the cutaneous infection was presented only by a single focus without spreading to other skin areas and mainly affected the arm or the face [6,16,17,18]. In addition, trauma to the affected area usually preceded the development of infection.

Diagnosis of mucormycosis requires a combined approach with the inclusion of imaging, histological and microbiological methods. The histopathological examination presents wide, coarse, and ribbon-like nonseptate hyphae. Angioinvasion and necrosis are often observed. When clinical samples are inoculated on agar media, visible fungal growth is detected in only 1/3 of the cases. Isolating the pathogen on artificial media is challenging because of the fragile nature of the hyphae, which are quickly destroyed when processing the material [2]. If successful, the cultivation of the molds on growth media can aid their identification and determination of antifungal susceptibility [19]. Furthermore, microscopic and macroscopic examination can distinguish the causative agents of mucormycosis from other molds also associated with invasive fungal infections, such as *Aspergillus* spp. and *Fusarium* spp. The coarse and nonseptate hyphae of the representatives of *Mucorales* spp. are easily differentiated from the narrow and septate hyphae of *Aspergillus* spp. and *Fusarium* spp. [15]. Additionally, the hyphae of the *Mucorales* spp. branch at a right angle (90°), while the hyphae of the other molds tend to branch at 45° [20]. In addition to the standard microbiological tests, molecular genetic methods such as PCR and DNA sequencing can be very helpful for diagnosis, leading to improved detection of the causative agents. These techniques can also be methods of choice for species identification by using genetic targets such as 18S ribosomal RNA and internal transcribed spacers (ITSs) [15]. Matrix-assisted laser desorption ionization-time of flight mass spectrometry (MALDI-TOF MS) is another method with great potential for fungal identification, but at the present time it is only limited to molds already isolated on growth media, and the available database needs to be improved and further expanded [15].

The serologic tests are another aspect of the complex diagnostic approach of the invasive fungal diseases. Various serum markers can be screened and monitored when invasive fungal infections occur with 1, 3-beta-D-glucan (BDG) and *Aspergillus* galactomannan being the most commonly used. However, these fungal cell components are not present in the *Mucorales* molds. Although negative in mucormycosis, when the positive result is obtained, this can rule out *Mucorales* infection. Currently, no serum markers are in use to diagnose mucormycosis [15].

Typical for the histological presentation of mucormycosis, we observed fat necrosis, leukocyte infiltrates, fungal angioinvasion, an abundance of nonseptate, and coarse branching at 90° mycotic hyphae. Mold growth was documented and detected on the SDA and DNA of clinically significant molds associated with mucormycosis.

Therapy of cutaneous mucormycosis is complex and usually includes surgical and antifungal treatment [2]. According to recent pediatric study [10], the combination therapy approach leads to a fatal outcome in 18.5% of cases, compared to 60% when only antifungal treatment is used. Multivariate analyses of studies, which include patients with hematological malignancies, show amphotericin B to be the agent of choice associated with a favorable outcome [21]. The newer triazoles, posaconazole and isavuconazole, can be alternatives to amphotericin B when stepdown therapy is needed or the patients cannot be treated with amphotericin B [22,23]. A drawback of these relatively new agents is the limited number of studies in the pediatric population [2]. It should be taken into consideration that *Mucorales* are naturally resistant to several antifungal agents, such as flucytosine, echinocandins (anidulafungin, micafungin, and caspofungin) and representatives of the azole group (fluconazole and voriconazole) [15]. Moreover, prophylaxis or treatment with some antifungals is identified as a predisposing factor for the development of mucormycosis. In their study, Kontoyiannis et al. followed patients with invasive mucormycosis [24]. Analyzing the obtained results, they reported that voriconazole use is an independent risk factor for the occurrence of mucormycosis in patients with hematological malignancies. A possible reason for this may be that the frequent use of voriconazole to prevent invasive fungal infection is caused by the more common fungal agents, such as *Candida* spp. and *Aspergillus* spp. in severely immunocompromised individuals, and the lack of activity of this agent against *Mucorales* molds.

However, if a good therapeutic response to amphotericin B monotherapy is not achieved, some authors recommend a combination of amphotericin B with azole anti-fungal agents (posaconazole or itraconazole). When the combination treatment is not effective, a salvage triple therapy including agents from three classes of antifungals (polyenes, echinocandines and azoles) can be initiated. There are no specific recommendations for the duration of mucormycosis treatment. The process can take months or even a year [19].

One of the most severe complications of cutaneous mucormycosis is the hematogenous dissemination of the pathogen, observed mainly in individuals with underlying diseases and various risk factors [25]. When dissemination occurs, mortality rates may reach up to 94% [16]. A specific and rarely observed feature of the presented clinical case is that despite the presence of several significant risk factors (young age, acute lymphoblastic leukemia, chemotherapy, neutropenia) and the formation of multiple skin lesions, no dissemination of the pathogen and the involvement of the internal organs and central nervous system had developed.

## 4. Conclusions

To the best of our knowledge, this is the first report in Europe of primary multifocal cutaneous mucormycosis without systemic dissemination in a child with hematological malignancy. A rapid and complex diagnostic approach is of crucial importance for the timely initiation of an adequate therapy and successful management of this invasive fungal disease.

## Figures and Tables

**Table 1:** Laboratory blood test results at admission and during the hospital stay.

Laboratory Blood Test Results	At Admission	During Hospital Stay
Hemoglobin, g/L (130–180)	56	65–126
WBC, 10^9^/L (4–10)	5.7	0.2–10
Platelets, 10^9^/L (140–440)	19	7–324
Glucose, mmol/L (3.9–5.5)	4.9	4.5–9.1
Fibrinogen, g/L (2–4)	2.35	0.89–1.72

WBC—white blood count.

## Data Availability

Not applicable.
